# Meningiomas in Premenopausal Women: Role of the Hormone Related Conditions

**DOI:** 10.3389/fonc.2020.556701

**Published:** 2020-12-11

**Authors:** Francesco Maiuri, Giuseppe Mariniello, Teresa Somma, Elia Guadagno, Sergio Corvino, Serena Pagano, Valentina Orlando, Marialaura Del Basso De Caro

**Affiliations:** ^1^ Neurosurgical Clinic, Department of Neurosciences and Reproductive and Odontostomatological Sciences, University “Federico II”, Naples, Italy; ^2^ Section of Pathology, Department of Advanced Biomorphological Sciences, University “Federico II”, Naples, Italy

**Keywords:** meningioma, progesterone receptor expression, pregnancy, oral contraceptives, fertilization therapies

## Abstract

**Background:**

Several epidemiological and pathological findings suggest that the female sex hormones may influence the development of meningiomas. However, the role of pregnancy, oral contraceptives, and fertilization therapies is still controversial.

**Methods:**

From the surgical series of 354 patients with meningiomas operated between 2006 and 2019, the group of 72 premenopausal women was separately considered. The tumor location, WHO grade, Ki67-labeling index (LI), progesterone receptor (PR) expression, and histological types were studied in premenopausal women with and without hormone-related conditions were compared.

**Results:**

In this premenopausal group, 24 patients had hormone-related conditions, including use of oral contraceptives in 16, intrauterine fertilization in one, pregnancy in three, and tumors of the female reproductive system in four. The group of patients with hormone-related conditions, as compared to that with no hormone related conditions, showed slightly lower median age (38 *versus* 43 years) and no significant difference of meningioma location WHO grade, Ki 67-Li, PR expression and histological type. The clinical onset during pregnancy in three patients and tumor growth during contraceptive progesterone therapy in two others were evidenced.

**Conclusion:**

The biological behavior of meningiomas and their pathological findings, including PR expression, are not correlated with the different hormone related conditions in premenopausal female patients. Contraceptives and fertilization therapies, mainly with progesterone, should be avoided in patients with meningiomas.

## Introduction

Meningiomas are mostly benign tumors which arise from meningothelial cells of the arachnoid membrane; their incidence is about two fold higher in women than in men (1).

Several epidemiological and pathological findings other than the higher female incidence may suggest that sex hormones may play a role in the development of these tumors. These include the frequent presence of progesterone and estrogen receptors in the meningioma tissue (2–7), the possible association with tumors of the female system (8–10), the documented changes of the meningioma biology during the menstrual cycle and pregnancy (11–13), the sometimes reported regression after delivery (14), the *in vitro* proliferation of meningioma cell lines in culture after exposure of estrogen and progesterone (15, 16). Besides, the incidence and risk of meningioma in patients with sex hormone-related conditions and during the exogenous use of sex hormones for contraceptive therapies have been investigated in several studies (16–22).

In this monoinstitutional study we have investigated the epidemiological and pathological findings of premenopausal women with meningioma and the effects of the sex hormone-related conditions in this age group.

## Materials and Methods

### Patient Population and Study Design

Three hundred fifty-four patients with primary intracranial and spinal meningiomas who underwent surgery at the neurosurgical clinic of the “Federico II” University of Naples between January 2006 and April 2019 were retrospectively reviewed. Cases of recurrent meningiomas and those with insufficient data were excluded. From the overall series, 72 female patients where the diagnosis of meningioma was made in the premenopausal age period were selected for the study. These 72 patients were classified in two groups: group A with sex hormone-related conditions at the meningioma diagnosis and group B with no hormone-related conditions.

Ethical approval for this human study was not required according to local and or national legislation.

### Analyzed Factors

The factors analyzed in the study include meningioma location, WHO grade, progesterone receptor (PR) expression, Ki67-MIB1, recurrence rate. The sex hormone-related conditions, which were analyzed in the premenopausal group, include exogenous hormone contraceptive therapies, pregnancy at the meningioma diagnosis, hormone-related extraneural tumors, and fertilization therapies.

The tumor location was defined from the review of the MR images and the surgical descriptions. Four groups were identified: group 1 or medial skull base including olfactory groove, ethmoidal–sphenoidal planum, tuberculum sellae, parasellar, clival-petroclival, and foramen magnum meningiomas; group 2 or lateral skull base, including the middle and lateral sphenoid wing and temporal fossa meningiomas and those of the petrous bone and occipital fossa; group 3 or non-skull base, including convexity, parasagittal or falx meningiomas, those of the tentorium, cerebellar convexity, and pineal region, and those of the lateral ventricles; group 4, including spinal meningiomas.

The surgical specimens were reviewed independently by two pathologists (MC and EG). The WHO grade was defined according to the 2007 WHO classification (23). The immunohistochemical studies were performed to evaluate the Ki67-MIB1 and the PR expression. The specimens were fixed in neutral buffered 10% formalin, embedded in paraffin and cut into sections of 5 mm thickness.

**Figure 1:**
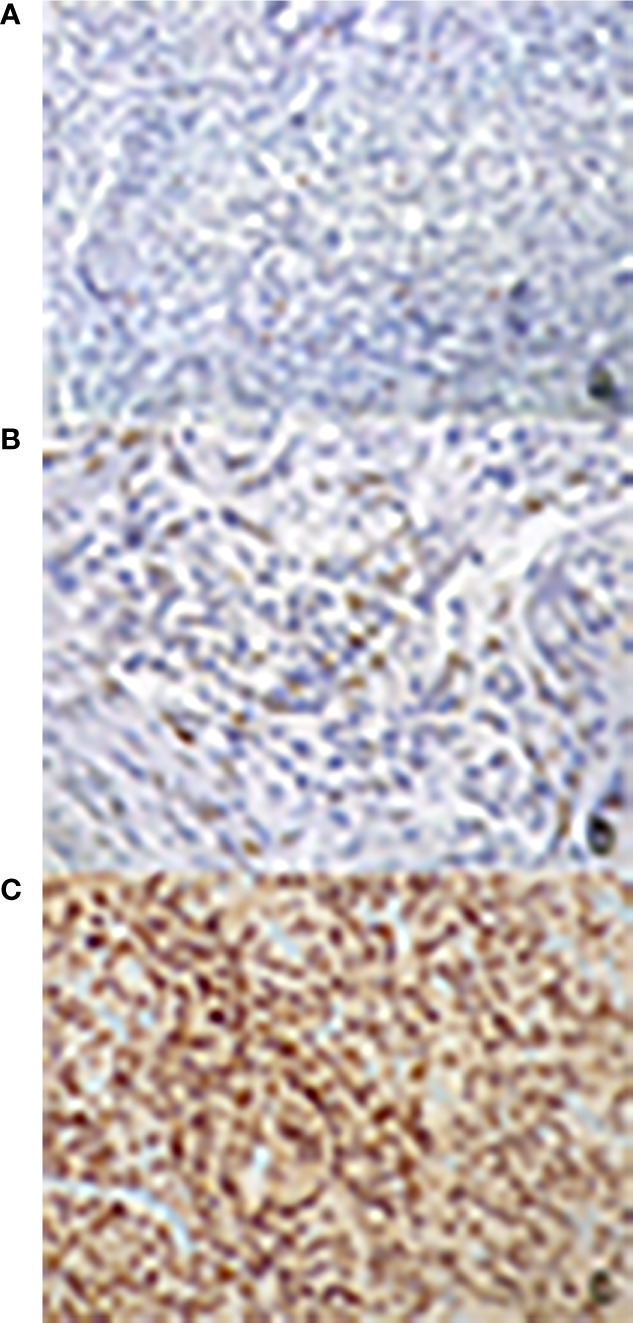
Immunohistochemical evaluation of progesterone receptor antibody expression: nuclear signal respectively in less than 1% **(A)**, in 15% **(B)** and in 95% **(C)** of neoplastic cells (200× magnification).

The expression of PR was determined in all specimens with monoclonal antibody against the progesterone (DAKO 1:400, overnight incubation). The quantitative evaluation was expressed as percentage for positive nuclei among 100 cells, for a total of 500 cells. The following score was used: 1. absent or low (L) (<15%); 2. moderately low (ML) (16–50%); 3. moderately high (MH) (51–79%); 4. high (H) (≥80%) (**Figure 1**).

The expression of Ki67-MIB1 was evaluated in all specimens by using the monoclonal antibody MIB1 Immunotech^®^ (DAKO system, dilution 1:1,000, overnight incubation). The streptavidin–biotin system and the diaminobenzidine (DAB) were used for antigen detection and visualization. A specimen of breast carcinoma was used as a positive control. Ki67-LI count was performed by eye counting, taking the average on five adjacent representative fields of neoplastic cells in a hot spot area. The values of Ki67-LI were classified into two groups: group I ≤4%; group II >4%.

The histological types of WHO grade I meningiomas were classified as: meningothelial, transitional, fibroblastic, psammomatous, microcystic, secretory, chordoid.

### Statistical Analysis

The meningioma location, WHO grade, Ki67-MIB1, PR expression and histological subtype were analyzed in all patients and stratified in the two groups, of patients with and without sex hormone-related conditions.

The data were analyzed by one-way ANOVA test or Fisher’s exact test, and p-value was correlated. A p value ≤0.05 was considered statistically significant. All tests were two-sided and carried out with Graph Prism 5 software (Graph Pad Software, La Jalla. CA. USA).

## Results

### Epidemiological and Pathological Data

The 72 female patients where the meningioma was diagnosed in the premenopausal age period account for 20% of the overall series of 354 meningiomas and 27.5% of the 262 female patients at all ages. Twenty-four patients (39.3%) were in group A with sex hormone-related conditions at the meningioma diagnosis and 48 (66.7%) in group B with no hormone-related conditions. The epidemiological and pathological data are summarized in **Table 1**. The patient age ranged from 19 to 52 years (median age 42 years), with no significant difference between groups A and B (38 *vs* 43 years, p = 0.81). According to the location (**Table 1**) 18 meningiomas (25%) were medial skull base, 11 (15%) lateral skull base, 40 (56%) non-skull base and three (4%) spinal. Fifty-five tumors (77%) were WHO grade I and 17 (23%) WHO grade II. The Ki-67Li was ≤4% in 43 patients (60%) and >4% in 29 (40%). The PR expression was <15% of the tumor cells in 10 cases (14%), between 16 and 50% in 17 (28%), between 51 and 79% in nine (13%) and ≥80% in 36 (50%). The more frequent histological subtypes of WHO I meningiomas were transitional (49%), fibroblastic (27%), and meningothelial (11%), whereas others were infrequent. The differences of tumor location, WHO grade, Ki67Li, PR expression, and histological subtype between group A and group B were not statistically significant (**Table 1**).

**Table 1:** Epidemiological and pathological data on premenopausal women with and without hormone-related conditions.

Covariates	Overall premenopausal A + B (72 pts)	Group A (24 pts)	Group B (48 pts)	Statistical significance P value
Median age	42 y	38 y	43 y	0.081
Meningioma location - Median skull base - Lateral skull base - Non-skull base - Spinal	18 (25%) 11 (15%) 40 (56%) 3 (4%)	8 (33%) 4 (17%) 12 (50%) -	10 (21%) 7 (15%) 28 (58%) 3 (6%)	
WHO grade - I - II	55 (77%) 17 (23%)	17 (70%) 7 (30%)	38 (79%) 10 (21%)	
Ki67Li - ≤4% - >4%	43 (60%) 29 (40%)	16 (67%) 8 (33%)	27 (56%) 21 (44%)	
Progesterone receptor expression - <15% - 16–15% - 51–79% - ≥80%	10 (14%) 17 (23%) 9 (13%) 36 (50%)	5 (21%) 6 (25%) 2 (8%) 11 (46%)	5 (10%) 11 (23%) 7 (15%) 25 (52%)	
Histological type (WHO I) - Meningothelial - Transitional - Fibroblastic - Psammomatosus - Microcystic - Secretory - Chordoid	6 (11%) 27 (49%) 15 (27%) 4 (7%) 1 (2%) 1 (2%) 1 (2%)	2 (12%) 10 (58%) 4 (24%) 1 (6%) - - -	4 (10.5%) 17 (45%) 11 (29%) 3 (8%) 1 (2.5%) 1 (2.5%) 1 (2.5%)	

### Sex Hormone-Related Conditions

**Figure 2:**
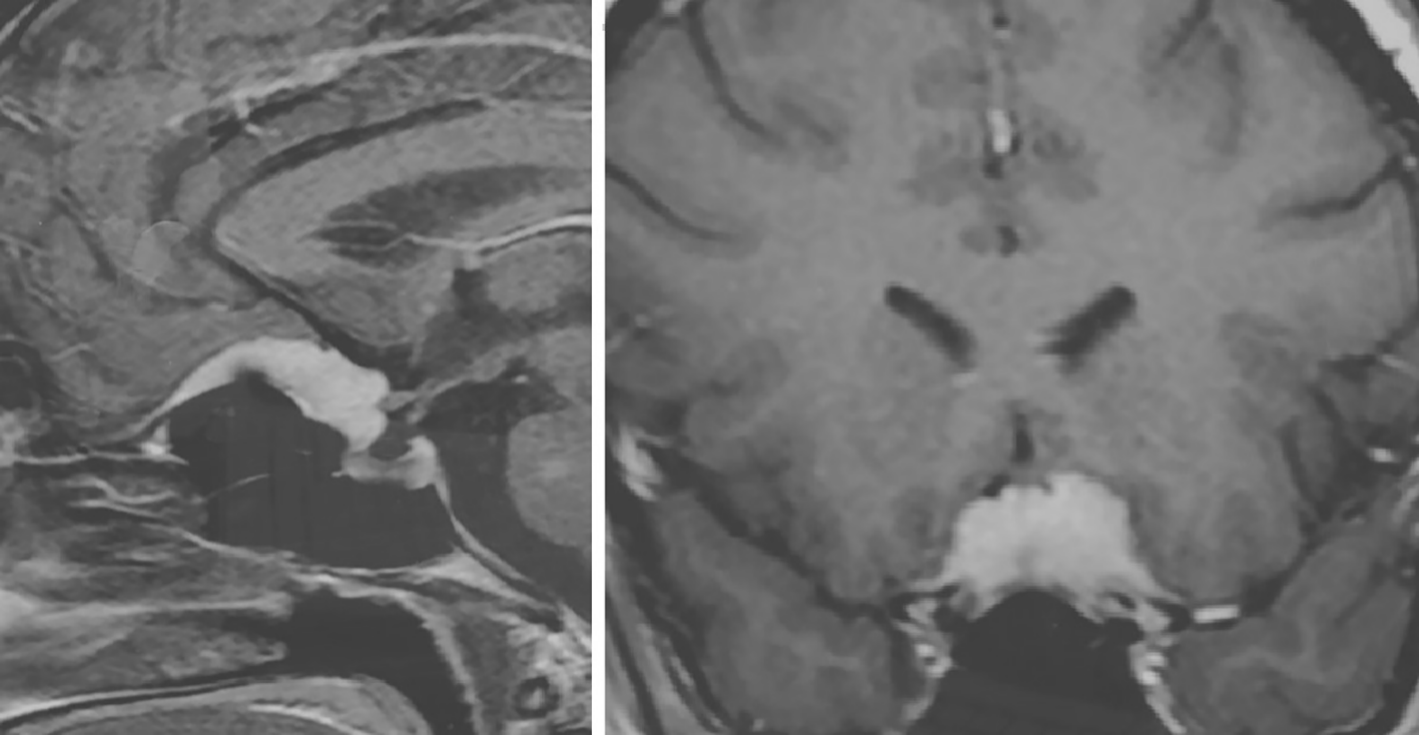
Post-contrast cranial MRI of 35-year-old female with tuberculum sellae meningioma presenting sudden onset of visual deficit during artificial *in vitro* fertilization treatment on the therapy with human chorionic gonadotrophin.

Twenty-four premenopausal women had associated sex hormone-related conditions at meningioma diagnosis. These include use of oral contraceptives in 16, assisted fertilization in one, pregnancy in three and hormone-related tumors of the sex female system in four (**Table 2**). Among the 16 patients with oral contraceptives (progesterone–estrogen), nine currently use the therapy at the meningioma diagnosis, and seven had used it up to 1 to 2 years before the diagnosis. Another patient, a 35-year-old woman with tuberculum sellae meningioma (**Figure 2**), presented visual deficit during the treatment of artificial *in vitro* fertilization and with human chorionic gonadotropin.

**Table 2:** Sex hormone-related conditions at meningioma diagnosis (24 patients).

•oral contraceptives •current use •previous use (1 to 2 years previously)	16 (67%) 9 7
•pregnancy	3 (12.5%)
•hormone-related tumors •breast cancer •ovarian cyst •ovarian adenoma	4 (16.5%) 2 1 1
•artificial fertilization	1 (4%)

Four patients had an associated tumor of the female system diagnosed and treated within 3 years before the meningioma diagnosis; these included ovarian cyst in one case, ovarian adenoma in another, and breast carcinoma in two.

Thirty-nine (54%) among the 72 patients (14 in group A and 25 in group B) experienced one or more previous pregnancies 3 or more years before the meningioma diagnosis. In these patients the previous pregnancy was not considered as hormone-related condition because the pregnancy-related hormonal effects were not present at the meningioma diagnosis. Thus, the correlation between pregnancy and meningioma occurrence and growth was difficult to be defined.

**Figure 3:**
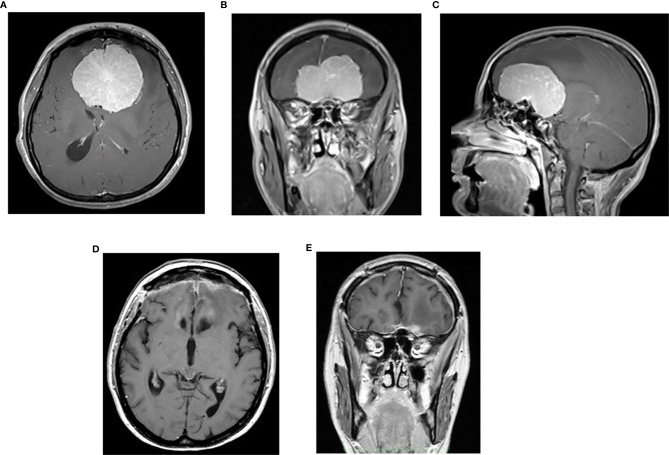
Preoperative post contrast MRI, axial **(A)**, coronal **(B)** and sagittal **(C)** sequences of 37-year-old female with large ethmoidal–sphenoidal planum meningioma presenting with visual loss and intracranial hypertension syndrome at 26^th^ week of pregnancy; postoperative post-contrast axial **(D)** and coronal **(E)** sequences.

**Figure 4:**
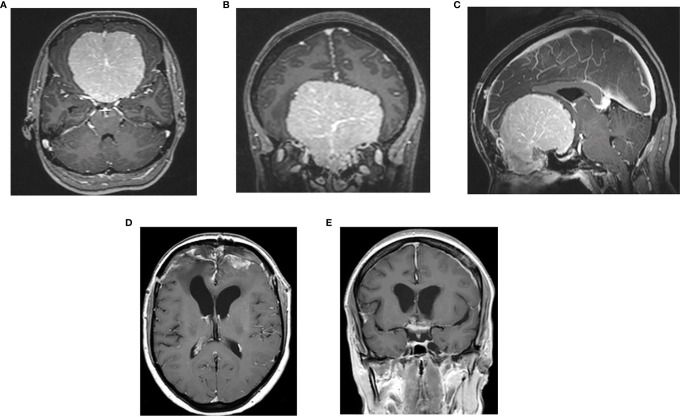
Preoperative post-contrast MRI, axial **(A)**, coronal **(B)** and sagittal **(C)** sequences of 30- year-old female with giant meningioma of spheno-ethmoidal planum presenting with bilateral visual deficit at 30^th^ week of pregnancy; postoperative postcontrast axial **(D)** and coronal **(E)** sequences: complete tumor removal.

In three women, the onset of the clinical symptoms occurred during pregnancy, between the 26^th^ and 30^th^ gestational weeks (**Table 3**). The tumor location was in all three cases on the midline skull base (ethmoidal–sphenoidal planum in two and tuberculum sellae in one); the tumor size was very large in two cases (6.5 and 7 cm) (**Figures 3**, **4**). A rapid decrease of the visual function was the presenting symptom in all three cases, with intracranial hypertension in one. Surgery for meningioma resection was performed 2 and 7 days after the delivery in two patients, whereas another decided to delay the operation. The tumor resection was complete (Simpson 2) (24). All three meningiomas were WHO grade I with high PR expression (≥80%) and Ki67-LI ≤4%. All three patients were symptom-free with no recurrence 18 months to 3 years after surgery.

**Table 3:** Data of three patients with meningiomas presenting during pregnancy.

N. of cases	A ge	Gestational age at onset (weeks)	Neurological symptoms	Delivery	Interval time between delivery and craniotomy	Meningioma location and size	Entity of resection	Pathology	Outcome
1.	37 y	26	intracranial hypertension sopor visual loss	cesarian delivery (32 wks)	2 days	ethmoidal–sphenoidal planum (6.5 cm)	complete (Simpson 2)	WHO I, transitional Ki67 3% PR 90%	cured at 6 y
2.	30 y	30	bilateral visual loss	spontaneous delivery (at term)	6 months	ethmoidal–sphenoidal planum (7 cm)	complete (Simpson 2) two stages	WHO I, transitional Ki67 3% PR 90%	cured at 26 mos
3.	2 8 y	28	bilateral visual loss	spontaneous delivery (at term)	7 days	tuberculum sellae (3 cm)	complete (Simpson 2)	WHO I, transitional Ki67 3% PR 80%	cured at 8 y

Two patients with known meningioma experienced tumor growth during contraceptive therapy with progesterone alone in a close MRI follow-up before surgery. The tumor location was parasagittal in one and tentorial in another; both had low PR values (30 and 1%).

Eight patients of the group of 72 premenopausal women experienced tumor recurrence (11%). It occurred in four cases (one WHO II and three WHO I) among 24 of group 1A with sex hormone-related conditions (16%) and in four cases (three WHO grade II and one WHO I) among 48 of group 1B with no hormone-related conditions (8%).

## Discussion

Meningiomas occur more frequently in postmenopausal women than in premenopausal ones, with a ratio of about 3:1 in our series. This difference is likely due to hormonal differences between the two age groups.

The role of the menopause as a risk factor for meningioma is cited in several studies, which provide controversial results. Some of them report a two to fivefolds higher risk (18, 25–28) or a moderately higher risk (29, 30) in postmenopausal women, whereas no association was found in others (31, 32). The occurrence of meningiomas in premenopausal women may involve some epidemiological and pathological aspects and may be influenced by coexisting hormone-related conditions, such as pregnancy and contraceptive and fertilization therapies.

We will discuss these conditions and the clinical significance of the correlated basic research studies.

### Meningioma Location and Pathological Findings

This study first discusses the location and pathological and immunohistochemical findings of meningiomas in premenopausal women.

No significant difference of tumor location was evidenced between premenopausal patients with and without hormone-related conditions. Although we have demonstrated in a previous study that medial skull base meningiomas have higher PR expression than lateral skull base and non-skull base ones (33), this finding is not correlated with the hormonal status.

We did not find significant differences for WHO grade, Ki 67-MIB1, and PR expression in premenopausal women, according to the presence of hormone-related conditions.

These data confirm that the biological behavior of meningiomas and their PR expression are not correlated with the different hormonal status of the female patients.

### Pregnancy

The relationship between pregnancy and meningiomas is a discussed problem in the neurosurgical practice. The risk of meningioma in female patients with one or more previous pregnancies does not seem to be increased (25–27, 30, 32, 34–37).

The onset of neurological symptoms of a known or a still undiagnosed meningioma during pregnancy (more often at the second or third trimester) or at delivery is a rare event which has been reported in 150 cases, as confirmed by several literature reviews (13, 38, 39).

Pregnancy-related meningiomas, when compared to those in the general population, are more frequently supratentorial (95%) and located in the medial skull base (68%); they are more often large (40% >5cm) and present in more than half of the cases with often rapid decrease of the visual function. Most reported cases are WHO grade I (75%), with mean Ki67-LI <4% and mean PR expression of 90%. The three cases of our series agree with these features.

Thus, pregnancy-related meningiomas exhibit more favorable pathological findings suggesting a better prognosis. The often reported sudden onset and rapid progression of clinical symptoms at the second or third trimester may depend on several factors, including increase of size, peritumoral edema, increase of the vascular supply to the tumor and probably pituitary-related hormone changes (13).

The rapid visual deterioration and sudden intracranial hypertension from a large meningioma, as in case one of our series, is a dangerous event at risk of visual deficit. The meningioma resection sudden after delivery is the best option, if possible. However, if necessary, an urgent craniotomy may be decided after the 27^th^ week, or the delivery may be anticipated to allow the craniotomy.

### Oral Contraceptives

The use of hormone-based contraceptives is widely diffuse in young women. The risk of meningioma correlated to contraceptive use has been discussed in several studies of the last 20 years (16–18, 20, 22, 26–28, 30, 32, 36, 37, 39). These provide controversial results, depending on several factors, such as type of contraceptive drugs, current or past use, and duration of the treatment.

The studies including patients using progesterone-only contraceptives (17, 18, 22) have shown increased risk of meningioma in those taking therapy for more than 5 years (17) and in those with PR positive meningiomas (18), and increased risk of recurrence and decrease of the progression free-survival (22). Two patients of our series who currently used progesterone-based contraceptives experienced tumor progression before surgical resection.

In the studies including patients who used estrogen-only or estrogen–progesterone contraceptives, those who currently used them showed increased risk of meningioma than those who had used them in the past (26, 27, 36, 39); besides, an increased meningioma risk was also evidenced for contraceptive use for more than 5 years (26, 27, 31).

The relationship between oral contraceptive use and hormone status of meningiomas is still unclear. Among the 16 patients of our series who used contraceptive therapy the PR expression of the meningioma was ≥80% in eight and <50% in eight, with no statistically significant difference. Korhonen et al. (18) report slightly higher risk for tumors expressing ER than for PR; on the other hand, Horland et al. (22) did not find significant differences. Custer et al. (26) showed increased risk of meningioma with low PR expression during contraceptive therapy. The two patients of our series showing increased tumor growth before surgery had low PR expression (30 and 1%). This agrees with the known more aggressive tumor biology in cases with low PR expression (40).

The experiences of our and other studies suggest avoiding contraceptive therapy, mainly with progesterone, in patients with meningioma.

### Fertility Therapies

The fertility treatments include a variety of methods: pharmacological ovarian stimulation, intrauterine insemination, *in vitro* fertilization, injection of human chorionic gonadotropin. One patient of our series with tuberculum sellae meningioma developed visual symptoms during the artificial insemination and treatment with chorionic gonadotropin.

The relationship between fertility treatment and meningiomas has scarcely been discussed. In the study of Korhonen et al. (18) the fertility treatments did not influence the risk of meningioma. Three single case reports describe meningiomas diagnosed in women with history of fertilization (41–43). In the study of Shahin et al. (44), the group of female patients with meningioma and history of fertility treatment had significantly younger age and higher rate of multiple non-skull base meningiomas as compared to the group with no fertility treatment. The development of meningiomas, even multiple, was reported in patients exposed to high-dose progesterone therapy (45, 46). All these data suggest that fertility treatments may influence the meningioma tumorigenesis. However, further studies are needed to better define this relationship.

## Conclusion

The pathological findings, biological behavior, and PR expression of meningiomas are not correlated with the hormone status and hormone-related conditions of the female patients. Pregnancy may be responsible for the sudden clinical onset of intracranial meningiomas because of the hormone-related tumor changes. Contraceptive and fertilization therapies, mainly with progesterone, should be avoided in patients with known meningiomas because of the risk of symptom occurrence and tumor progression.

### Characteristics of the Study

#### Strengths

This study discusses a scarcely focused aspect of meningiomas concerning their occurrence in premenopausal women and the differences between those with and without hormone-related conditions.

#### Limitations

This study is retrospective. Data on the hormonal substitution among postmenopausal women are lacking.

## Data Availability Statement

The original contributions presented in the study are included in the article/supplementary material. Further inquiries can be directed to the corresponding author.

## Author Contributions

All authors listed have made a substantial, direct, and intellectual contribution to the work and approved it for publication.

## Conflict of Interest

The authors declare that the research was conducted in the absence of any commercial or financial relationships that could be construed as a potential conflict of interest.

## Abbreviation

Ki67-LI, Ki67 Labelling Index; PR, Progesterone Receptor; ER, Estrogen Receptor; MRI, Magnetic Resonance Images.
